# TEG Platelet Mapping and Impedance Aggregometry to Predict Platelet Transfusion During Cardiopulmonary Bypass in Pediatric Patients

**DOI:** 10.3389/fped.2019.00509

**Published:** 2019-12-12

**Authors:** Erin E. Barker, Arun Saini, Avihu Z. Gazit, Susan M. Shea, Sirine Baltagi, Brian F. Gage, Philip C. Spinella

**Affiliations:** ^1^Division of Pediatric Critical Care, University of Rochester, Rochester, NY, United States; ^2^Division of Pediatric Critical Care, Washington University School of Medicine, St. Louis, MO, United States; ^3^Division of Pediatric Critical Care, Baylor College of Medicine, Texas Children's Hospital, Houston, TX, United States; ^4^Department of Critical Care Medicine, Saint Joseph Children's Hospital, Tampa, FL, United States; ^5^Division of General Medical Sciences, Washington University School of Medicine, St. Louis, MO, United States

**Keywords:** thromboelastography, congenital heart disease, post-operative hemorrhage, impedance aggregometry, cardiopulmonary bypass

## Abstract

**Background:** Cardiopulmonary bypass-related platelet dysfunction can increase the risk of intra- and post-operative bleeding in children undergoing cardiac surgery. More accurate laboratory tests that identify acquired platelet abnormalities could allow for rapid identification of patients at risk of bleeding and provide therapies that could reduce bleeding and platelet transfusions. We hypothesized that thromboelastography with platelet mapping (TEG-PM) and multiple electrode impedance aggregometry (MEIA) as functional measures of platelet function would predict who will require platelet transfusion. Our secondary hypothesis was that platelet aggregation at both arachidonic acid (AA) and adenosine diphosphate (ADP) receptors would correlate between TEG-PM and MEIA results.

**Methods:** In this prospective study from August 2013 to December 2015, children from newborn to 5 years of age with congenital heart disease undergoing cardiopulmonary bypass had blood samples collected and analyzed at four time points: pre-bypass, post-bypass, post-operatively on arrival to the Cardiac Intensive Care Unit, and 24 h after arrival.

**Results:** Of the 44 patients analyzed, the 10 patients who received peri-operative platelet transfusion were significantly younger (*p* = 0.05), had higher STAT (Society of Thoracic Surgeons-European Association for Cardio-Thoracic Surgery) Mortality Categories (*p* < 0.002) and longer cardiopulmonary bypass times (*p* = 0.02). In univariate analysis, four variables were associated with peri-operative platelet transfusion: pre-operative age [OR 0.95 (0.93, 0.98), *p* = 0.03], cardiopulmonary bypass time [1.5 (1.31, 1.68), *p* = 0.008], STAT Mortality Category [3.64 (3.40, 3.87), *p* < 0.001], and TEG-PM ADP [0.79 (0.65, 0.93), *p* = 0.04]. ROC analysis demonstrated moderate predictive value of TEG-PM ADP with AUC of 0.745 (0.59, 0.91). A TEG-PM ADP value of less than or equal to 21 had 85% sensitivity and 70% specificity for platelet transfusion. In the multivariate analysis, only STAT Mortality Category predicted platelet transfusion. TEG-PM and MEIA results correlated for the AA receptor at all 4 time points, but the same tests at the ADP receptors did not correlate.

**Conclusions:** TEG-PM ADP may provide more clinically relevant information regarding platelet function compared to the MEIA at the ADP receptor in children requiring cardiopulmonary bypass. There was limited correlation between TEG-PM and MEIA results which raises a concern about the accuracy of these tests at the ADP receptor. Lower pre-operative TEG-PM ADP MA may predict intra-operative platelet transfusions; however, larger studies are needed to determine the utility of TEG-PM and MEIA in guiding platelet transfusions in this population.

## Introduction

Cardiopulmonary bypass in pediatric congenital heart disease operations causes platelet dysfunction due to shear stress, changes in receptor expression, and platelet exhaustion due to activation (1). These acquired platelet abnormalities increase the risk of post-operative bleeding and transfusion. In the peri-operative phase, platelet count coupled with clinical data are used to determine administration of platelets. Unfortunately, in the absence of bleeding, platelet count alone is not sufficient to determine risk of bleeding since it does not address platelet function (2). In a retrospective study examining bleeding in pediatric patients support on extracorporeal membrane oxygenation, there was no significant difference in platelet counts between the patients with severe bleeding and those without (3). Functional measures of hemostasis such as thromboelastography (TEG, Haemonetics Corp., Braintree, MA) and Multiple Electrode Impedance Aggregometry (MEIA, Verum Diagnostica, Roche, Munich, Germany) may be able to identify acquired platelet abnormalities and allow for goal-directed therapies that could reduce bleeding and the need for platelet and other blood product transfusions. Furthermore, these tests are available as point-of-care tests allowing rapid results and treatment of acquired platelet abnormalities. Reducing exposure to blood products is important since it may improve outcomes (4).

TEG measures viscoelastic changes during clot initialization, formation, and lysis (5). TEG with platelet mapping (TEG-PM), a modification of the standard TEG, evaluates platelet function through direct activation of arachidonic acid (AA) and adenosine diphosphate (ADP) receptors, with analysis of the resulting formation of TEG curves. Comparison of these curves with the standard kaolin activated TEG assay allows for the quantification of platelet aggregation and inhibition at both AA and ADP receptors (5). Platelet aggregration results are reported as a maximum amplitude which refers to the highest point of the TEG curve and inhibition is reported as a percent (5). MEIA is a point-of-care test approved for clinical use that evaluates platelet function which measures the change in impedance due to aggregate formation in response to an agonist (6). There are many agonists available, including ADP and AA.

Though ADP and AA receptors are weak agonists for platelet aggregation, they are essential in amplification and propagation of initial platelet aggregation in response to either collagen or thrombin. Inhibition of either one or both may prevent the formation of an effective hemostatic clot promptly. Platelets on activation secret stored ADP from dense granules to further activate and recruit new platelets flowing in the vicinity of the initial clot formation. Thromboxane A2 is produced by cyclooxygenase (COX-1) enzyme in the platelets in response to AA-mediated activation. Thromboxane A2 further increases platelet aggregation in response to collagen/thrombin and enhances the activation of new platelets in the vicinity. Thromboxane A2 has a limited paracrine effect due to its very short half-life (about 30 s).

There have been many attempts to predict post-operative bleeding with classic coagulation tests, viscoelastic tests, and MEIA following cardiopulmonary bypass with mixed results (7–13). To the best of our knowledge, there have been no studies attempting to predict peri-operative bleeding in pediatric cardiac patients combining TEG-PM and MEIA. We hypothesized that TEG-PM and MEIA as measures of platelet function can predict platelet transfusion in children undergoing repair of congenital heart disease using cardiopulmonary bypass. There have been variable results in studies evaluating TEG-PM and/or MEIA, but we hypothesize that in combination, these assays will predict patients who will require platelet transfusion. Our secondary hypothesis was that platelet aggregation at both AA and ADP receptors on TEG-PM would correlate with MEIA results at the same receptors. There have been no studies to date evaluating the correlation between these two tests in children undergoing repair of congenital heart disease using cardiopulmonary bypass.

## Materials and Methods

### Study Design

This prospective, observational study was approved by the Institutional Review Board at Washington University School of Medicine in St. Louis, MO (201302093). Between August 2013 and December 2015, newborns to 5-year-old patients with congenital heart disease who underwent repair using cardiopulmonary bypass and with moderate to severe surgical complexity were eligible. Exclusion criteria were weight <3.5 kg, mild surgical complexity, ECMO/mechanical circulatory support before or after the procedure, and underlying bleeding disorder. All eligible patients were identified and their parents consented in writing during the pre-operative visit. Anesthesia and post-operative care were carried per usual practice.

### Blood Sampling

Blood samples were collected for each patient at four time points: following anesthesia induction, following removal from cardiopulmonary bypass, post-operatively on arrival to the Cardiac Intensive Care Unit (CICU), and 24 h after arrival to the CICU. All samples were obtained from indwelling catheters.

### Clinical Data

STAT (Society of Thoracic Surgeons-European Association for Cardio-Thoracic Surgery) Mortality Categories, which are a validated measure of cardiac lesion complexity, were recorded. The STAT Mortality Categories are scores from 1 to 5 assigned to each congenital heart disease procedure based on predicted mortality (13). We collected clinical data, including blood product transfusion, chest tube output, urine output, treatment with medications post-operatively (including vasoactive drugs), and clinically evident thrombosis. To estimate significant clinical bleeding, we use peri-operative platelet transfusion as it is our institutional practice to transfuse platelets with significant clinical bleeding. Platelet transfusions ranged from 10 to 15 mL/kg. Platelet transfusions occurred either intraoperatively or postoperatively. Our institution did not have a specific platelet transfusion guideline for cardiac intensive care unit. Intraoperative transfusions were given based upon the rough estimate of ongoing surgical site bleeding by the operating surgeons. Platelet count was not routinely measured intra-operatively for clinical purposes. The postoperative platelet transfusions were given based on bleeding from chest tubes and platelet count though no specific platelet count value was used to guide transfusion.

### Laboratory Analysis

At each of the four time points, the following laboratory analyses were done: TEG-PM, MEIA, prothrombin time (PT), international normalized ratio (INR), activated partial thromboplastin time (PTT), fibrinogen, and complete blood count (CBC). Except for MEIA, all laboratory analyses were done in the Core Lab at St. Louis University Children's Hospital per their usual protocol. Samples were transported immediately after being drawn to the Core Lab and run within 1 h. TEG-PM samples were collected in sodium citrate tubes. MEIA samples were collected in hirudin tubes and were run within 1 h of collection on the Multiplate® Analyzer (Verum Diagnostica, Roche, Munich, Germany). Quality controls and recalibration were done per manufacturer's recommendations. AA and ADP were tested on all samples and increases in impedance are expressed as area under the curve (AUC) units (collagen is also an available agonist but was not tested due to space limitations).

### Statistical Analysis

Prior to analysis, we excluded one subject during the pre-operative period because the results were outliers (>3 SD above mean). All of the statistical analysis was performed using either IBM SPSS Statistics (version 23 and 25, 2015) or R (version 3.4.0). Continuous non-parametric data were compared using Mann-Whitney *U*-Test and Wilcoxon signed-rank test, as appropriate. Fisher's exact test was used for categorical variables. To investigate factors associated with platelet transfusion during the peri-operative period, data were analyzed using both univariate and multivariate logistic regression. Our focal variables in these analyses were TEG-PM and MEIA for ADP and AA receptors. We also tested for associations between platelet transfusion and weight (kg), cardiopulmonary bypass and aortic cross clamp duration (mins), STAT Mortality Categories, and type of congenital heart disease (cyanotic or acyanotic). Associations were evaluated using likelihood-ratio tests or Fisher's exact tests. To determine independent associations, all variables that were significant (two-sided, *p* < 0.05) were considered. Due to the low number of patients receiving platelet transfusions, we were not able to include all potential confounders. Since our hypothesis included TEG-PM ADP, we analyzed this variable in a logistic regression model that adjusted for the covariate that was most strongly associated with platelet transfusion (STAT Mortality Category). Data are presented as median (IQR) and odds ratio (95% CI). A receiver operating characteristic curve (ROC) was created to establish predictive models for platelet transfusion based on pre-operative platelet count, TEG-PM ADP and AA as well as MEIA ADP and AA. The correlation between MEIA and TEG-PM AA and TEG-PM ADP was assessed using linear regression.

## Results

Forty-seven patients were enrolled in the study. Three patients were excluded following enrollment: one patient did not require cardiopulmonary bypass; the second required post-operative extracorporeal membrane oxygenation support; and the third was enrolled in another study limiting our ability to obtain the needed blood samples. Thus, 44 patients were included in the statistical analyses. Their demographics are displayed in Table 1.

**Table 1:** Patient demographics.

**Variable**	**Patients not requiring platelet transfusion (*n* = 34)**	**Patients requiring platelet transfusion (*n* = 10)**	***P*-value**
Age (months), median (IQR)	16 (6, 42)	6 (3, 12)	0.05
Gender: no (%)			
Male	19 (55.9%)	8 (80%)	0.27
Female	15 (44.1%)	2 (20%)	
Weight (kg), median (IQR)	9 (5.8, 17.7)	7.4 (4.9, 9.9)	0.16
BSA (m^2^), median (IQR)	0.42 (0.30, 0.60)	0.38 (0.29, 0.43)	0.20
Race: no (%)			
Caucasian	28 (82.4%)	9 (90%)	1
African American	2 (5.9%)	0	
Other	4 (11.8%)	1 (10%)	
Type of CHD: no (%)			
Cyanotic	17 (50%)	7 (70%)	0.31
Acyanotic	17 (50%)	3 (30%)	
STAT mortality category: no (%)			
1	21 (61.8%)	0	<0.0001
2	8 (23.5%)	1 (10%)	
3	4 (11.8%)	2 (20%)	
4	1 (2.9%)	3 (30%)	
5	0	4 (40%)	
CPB time (minutes), median (IQR)	82 (59, 109)	125 (94, 141)	0.02
Aortic cross clampTime (minutes), median (IQR)	41 (26, 60)	52 (21, 89)	0.4
Pre-operative platelet count, median (IQR)	291 (250, 325)	294 (231, 344)	0.89
Chest tube output (mL/Kg) in the 4 h following the procedure, median (IQR)	5.20 (3.05, 6.70)	12.45 (6.40, 25.52)	0.002
**Intra-operative blood product transfusion**			
Red blood cells, no (%)	22 (64.7%)	9 (90%)	0.12
Fresh Frozen Plasma, no (%)	22 (64.7%)	8 (80%)	0.31
Cryoprecipitate, no (%)	1 (2.9%)	4 (40%)	0.007
**Post-operative blood product transfusion**			
Red blood cells, no (%)	7 (20.6%)	4 (40%)	0.2
Fresh Frozen Plasma, no (%)	0	2 (20%)	0.05
Cryoprecipitate, no (%)	0	1 (10%)	0.23

*BSA, body surface area; CHD, congenital heart disease; CPB, cardiopulmonary bypass. Data presented as either a percentage (%) or as median (interquartile range)*.

Only 10 patients received peri-operative platelet transfusions (including one was transfused both intra- and post-operatively). Eight patients (18.2%) received intra-operative platelet transfusion and three patients (6.8%) received post-operative platelet transfusion. Comparison of patients who received peri-operative platelet transfusion with those that did not, revealed a significant difference in age (*p* = 0.05), STAT Mortality Category (*p* < 0.002), and cardiopulmonary bypass time (*p* = 0.02) (Table 1). Patients who received platelet transfusions also had increased chest tube output (*p* = 0.002). There was no difference in pre-operative platelet count, weight, body surface area (BSA, m^2^), type of congenital heart disease, or aortic cross clamp time among patients who received transfusions and those that did not.

**Figure 1:**
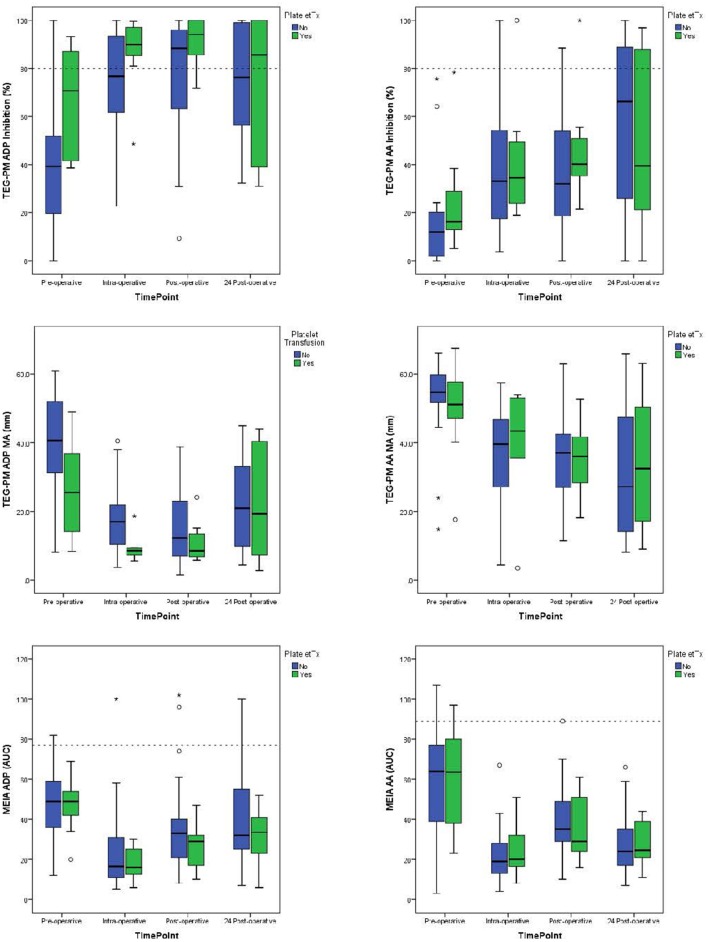
Median values for TEG-PM Inhibition, TEG-PM MA, and MEIA at the ADP and AA receptors for each time point. The blue bars represent test results for patients who required platelet transfusion and the green bars represent patients who did not require platelet transfusion. Dashed reference lines on inhibition graphs signifying significant inhibition at 80%. Reference line on MEIA graph signifies 10% of the reference range for children 1–4 years of age (14). TEG-PM, thromboelastography with platelet mapping; MA, maximum amplitude; ADP, adenosine diphosphate receptor; AA, arachidonic acid receptor; MEIA, multiple electrode impedance aggregometry; AUC, area under the curve. °Outliers (1.5–3x IQR); *Extreme outliers (>3x IQR).

The laboratory values demonstrate decreased platelet function following cardiopulmonary bypass when compared with pre-operative values. For patients not requiring transfusion (Table 2), there was a decrease in median TEG-PM MA results at both the AA and ADP receptors (*p* < 0.005 and *p* < 0.005, respectively) as well as median MEIA results for both the AA and ADP receptors (*p* < 0.005 and *p* = 0.006, respectively). There was also a significant increase in inhibition at both AA and ADP receptors (*p* = 0.006 and *p* < 0.005, respectively). This change was significant at all three time points when compared to their pre-operative values. For patients receiving platelet transfusion, the same pattern existed (Figure 1), but failed to reach significance except for MEIA AA and ADP receptors (*p* = 0.05 and *p* = 0.006, respectively). This may be due to the small number of patients that required platelet transfusion or even variability introduced with transfusions.

**Table 2:** Laboratory values according to time point and platelet transfusion.

**Laboratory test**	**Time point**	**No platelet transfusion**		**Platelet transfusion**	
		**Median (IQR)**	***P*-value**	**Median (IQR)**	***P*-value**
TEG-PM ADP MA (mm)	Pre-operative	43.8 (33.1, 55.2)	<0.005	17.5 (9.7, 42.0)	0.6
	Intra-operative	13.4 (7.5, 31.6)		8.0 (6.0, 16.1)	
	Post-operative	17.8 (7.1, 29.8)		12.1 (6.9, 21.9)	
	24 h post-operative	26.8 (10.1, 34)		16.1 (3.7, 36.8)	
TEG-PM AA MA (mm)	Pre-operative	56.6 (52.1, 60.4)	<0.005	44.9 (23.3, 63.1)	0.82
	Intra-operative	42.1 (30.4, 50.5)		39.5 (11.5, 51.4)	
	Post-operative	39.1 (26.6, 48.6)		39.2 (30.5, 50.3)	
	24 h post-operative	25.2 (14.2, 34)		36.7 (12.5, 60)	
TEG-PM ADP percent inhibition	Pre-operative	39 (16.6, 55.8)	<0.005	78.5 (39.4, 88.6)	0.114
	Intra-operative	81.3 (59.1, 95)		93 (72.8, 98.3)	
	Post-operative	85 (53, 99.1)		94.1 (82.2, 100)	
	24 h post-operative	69.8 (57.2, 98.6)		85.7 (40.9, 100)	
TEG-PM AA percent inhibition	Pre-operative	12 (3.6, 21.9)	0.006	16.1 (11, 48.4)	0.08
	Intra-operative	26.7 (15.8, 48.6)		39.8 (25.7, 65.3)	
	Post-operative	32 (17.4, 54.5)		43.5 (32.6, 66.6)	
	24 h post-operative	69.8 (25.7, 88.9)		52.7 (16, 90.2)	
MEIA ADP (AUC)	Pre-operative	45 (33, 57.75)	0.006	48 (34, 54)	0.006
	Intra-operative	17.5 (10.3, 37.8)		16 (11, 26)	
	Post-operative	35.5 (24.3, 53.3)		29 (16, 32)	
	24 h post-operative	37.5 (26.3, 58)		36 (31, 41)	
MEIA AA (AUC)	Pre-operative	59 (41, 68.5)	<0.005	45 (32, 74)	0.05
	Intra-operative	19.5 (11.3, 37.3)		20 (15, 34)	
	Post-operative	38 (30.5, 49.8)		31 (27, 58)	
	24 h post-operative	23.5 (17, 34.5)		26 (21, 39)	

*CPB, cardiopulmonary bypass; TEG-PM, thromboelastography with platelet mapping; ADP, adenosine diphosphate receptor; AA, arachidonic acid receptor; MEIA, multiple electrode impedance aggregometry; AUC, area under the curve*.

**Figure 2:**
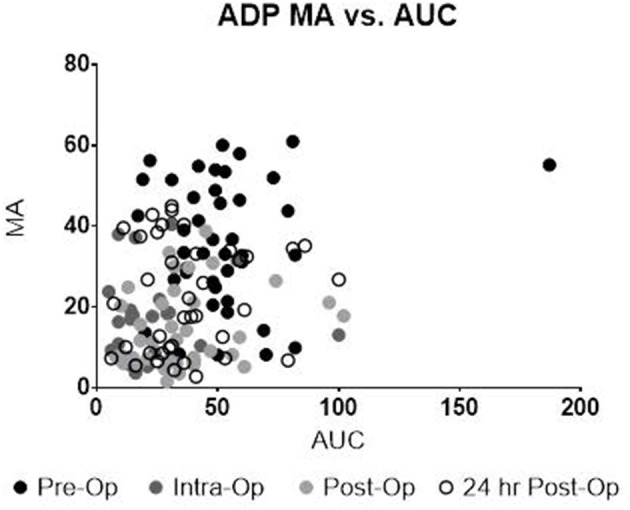
TEG-PM MA vs. MEIA AUC for the ADP Receptor at all sampling time points. TEG-PM MA, thromboelastography with platelet mapping maximum amplitude; ADP, adenosine diphosphate receptor; MEIA, multiple electrode impedance aggregometry; AUC, area under the curve.

**Figure 3:**
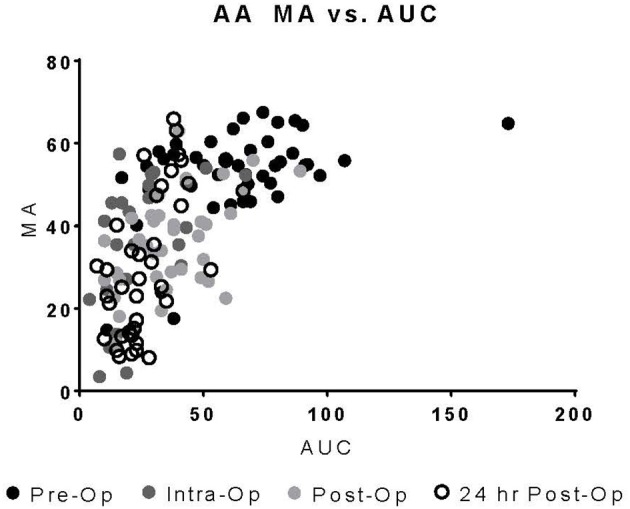
TEG-PM MA vs. MEIA AUC for the AA Receptor at all sampling time points. TEG-PM MA, thromboelastography with platelet mapping maximum amplitude; AA, arachidonic acid receptor; MEIA, multiple electrode impedance aggregometry; AUC, area under the curve.

Upon univariate analysis, pre-operative age, cardiopulmonary bypass time, STAT Mortality Category, and TEG-PM ADP, significantly predicted platelet transfusion (Table 3). Multivariate analysis indicated that TEG-PM ADP was not independently associated with platelet transfusion (Table 4). When evaluating the correlation between TEG-PM and MEIA results, the correlation was significant for the AA receptor for all time points; however, at the ADP receptor there was no correlation between the two tests (Table 5 and Figure 2, Table 6 and Figure 3).

**Table 3:** Univariate analysis predicting platelet transfusion.

**Clinical factors**	**Odds ratio (95% CI)**	***P*-value**
Age (months)	0.95 (0.93, 0.98)	0.03
Weight (kg)	0.98 (0.78, 1.18)	0.16
Cyanotic heart disease	0.82 (0.58, 1.05)	0.28
CPB time (per 10 min)	1.5 (1.31, 1.68)	0.008
STAT mortality category 4 or 5	3.64 (3.40, 3.87)	<0.001
Platelet count	0.88 (0.58, 1.17)	0.58
Pre-operative labs^*^		
TEG-PM ADP (mm)	0.79 (0.65, 0.93)	0.04
TEG-PM AA (mm)	0.84 (0.57, 1.11)	0.41
MEIA ADP (AUC)	0.99 (0.81, 1.15)	0.92
MEIA AA (AUC)	0.98 (0.86, 1.10)	0.83

* *Pre-operative labs drawn after induction of anesthesia. CPB, cardiopulmonary bypass; STAT, Society of Thoracic Surgeons-European Association for Cardio-Thoracic Surgery; TEG-PM, thromboelastography with platelet mapping; ADP, adenosine diphosphate receptor; AA, arachidonic acid receptor; MEIA, multiple electrode impedance aggregometry; AUC, area under the curve*.

**Table 4:** Multivariate analysis predicting platelet transfusion.

**Predictors of platelet transfusion**	**Odds ratio (95% CI)**	***P*-value**
STAT mortality category	55.30 (4.5, 981.1)	0.002
Pre-operative TEG-PM ADP	0.98 (0.91, 1.05)	0.52

*STAT, Society of Thoracic Surgeons-European Association for Cardio-Thoracic Surgery; TEG-PM ADP, Thromboelastography with platelet mapping at the adenosine diphosphate receptor*.

**Table 5:** Correlation between TEG-PM ADP MA and MEIA ADP AUC.

**Time point**	**R^**2**^**	***P*-value**
Pre-operative	0.102	0.52
Intra-operative	0.004	0.77
Post-operative	0.057	0.14
24 h post-operative	0.012	0.51

*TEG-PM, thromboelastography with platelet mapping; ADP, adenosine diphosphate receptor; MEIA, multiple electrode impedance aggregometry; AUC, area under the curve*.

**Table 6:** Correlation between TEG-PM AA MA and MEIA AA AUC.

**Time point**	**R^**2**^**	***P*-value**
Pre-operative	0.22	0.002
Intra-operative	0.26	0.009
Post-operative	0.27	0.0008
24 h post-operative	0.38	<0.0001

*TEG-PM, thromboelastography with platelet mapping; AA, arachidonic acid receptor; MEIA, multiple electrode impedance aggregometry; AUC, area under the curve*.

**Figure 4:**
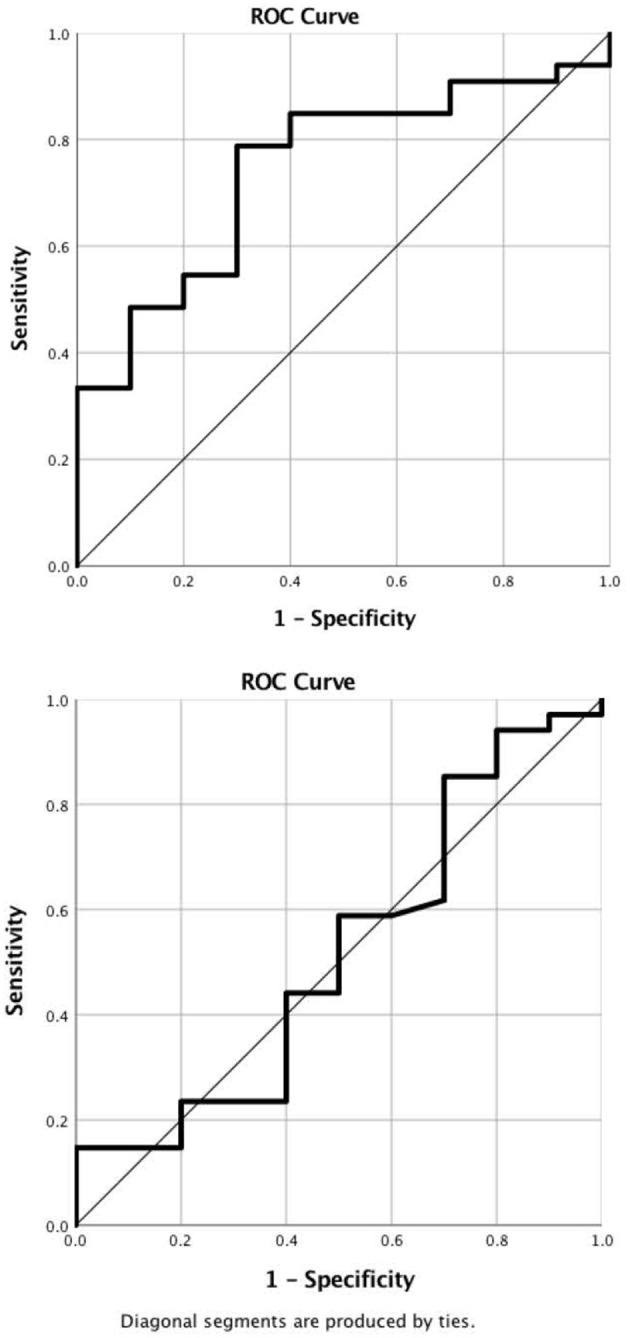
ROC Curves. **(A)** Pre-operative TEG-PM ADP demonstrates moderate predictive value for platelet transfusion. AUC is 0.75 (0.59, 0.91) with a *p*-value of 0.02. A cut off value of less than or equal to 21 mm has 85% sensitivity and 70% specificity for predicting platelet transfusion. **(B)** Platelet count. Pre-operative platelet count failed to predict platelet transfusion. AUC is 0.52 (0.30, 0.73) with a *p*-value of 0.88. TEG-PM ADP, thromboelastography with platelet mapping adenosine diphosphate receptor maximum amplitude.

In the ROC analysis, TEG-PM ADP demonstrated moderate predictive ability with AUC of 0.745 (95% CI 0.587–0.907) and *p*-value of 0.02 (Figure 4). A cut off value of less than or equal to 21 mm had a 85% sensitivity and 70% specificity for platelet transfusion. Platelet count, TEG-PM ADP, MEIA ADP and MEIA AA were not predictive of platelet transfusion.

## Discussion

This is the first study to combine TEG-PM and MEIA results to predict platelet transfusion in children undergoing repair of congenital heart disease with cardiopulmonary bypass. Similar to others, we found that there is a significant reduction in platelet function with cardiopulmonary bypass that is quantified by both TEG-PM and MEIA (9, 10). Although pre-operative TEG-PM ADP predicted platelet transfusion in univariate analysis and ROC analysis, it was not significant in the multivariate model.

There have been several adult studies that have utilized TEG-PM and MEIA to predict post- operative bleeding with conflicting results. Recently, Agarwal et al. prospectively studied 54 adults undergoing cardiac surgery (7). They demonstrated significant platelet inhibition on both TEG-PM and MEIA following cardiopulmonary bypass. However, neither of these assays correlated with post-operative blood loss. In another study, Petricevic et al. used thromboelastometry and MEIA to predict excessive post-operative bleeding in 148 adults undergoing elective cardiac surgery (15). They found significant differences at all three time points for the MEIA and thromboelastometry results between the “bleeders” and “non-bleeders.” The differences in the results between these two studies may be due to the larger sample size in the Petricevic study, or perhaps the difference in outcomes measured in the two studies.

There have also been two studies that evaluated prevention of post-operative bleeding using TEG- and MEIA-based guidelines. Weber at al. randomized 100 adults to conventional care vs. use of point-of-care TEG and MEIA to guide transfusions (11). This trial showed significant decrease in the amount of red blood cells, fresh frozen plasma, and platelets transfused in the point of care group. There have been few pediatric studies of thromboelastography. Saini et al. demonstrated severe platelet dysfunction in patients on extracorporeal membrane oxygenation (3). In a retrospective review of 150 children with congenital heart disease requiring cardiopulmonary bypass, Kane et al. demonstrated a reduction in platelet and cryoprecipitate transfusions when intraoperative TEG was used to guide transfusion (12).

Interestingly, our data indicate that there is a moderate correlation at the AA receptor between TEG-PM and MEIA but a poor correlation at the ADP receptor. This is consistent with the results George et al. found in adult trauma patients (16). This raises the question of which test is more accurate or clinically relevant at the ADP receptor. Our study suggests TEG-PM ADP might be more clinically relevant since pre-operative TEG-PM ADP on univariate analysis was predictive of peri-operative bleeding and platelet transfusion. Multivariate analysis did not result in similar findings, therefore additional data are required to determine which test, if any, can determine clinically relevant platelet dysfunction.

Because of the small sample size and the low number of patients receiving platelet transfusions, this study was underpowered to quantify the putative association between functional measures of platelet function and subsequent platelet transfusion. Even so, pre-operative TEG-PM ADP predicted platelet transfusion in the univariate analysis. A larger study is needed to either confirm or refute this relationship.

## Conclusion

TEG-PM ADP MA may provide more clinically relevant information regarding platelet function compared to the MEIA at the ADP receptor in children undergoing congenital heart disease operations with cardiopulmonary bypass. The change in correlation over time at the AA receptor between platelet function testing methods suggests post-operative acquired change in platelet function. Larger studies are needed to determine the utility of TEG-PM and MEIA in guiding platelet transfusions in this population.

## Data Availability Statement

The datasets generated for this study are available on request to the corresponding author.

## Ethics Statement

The studies involving human participants were reviewed and approved by Institutional Review Board at Washington University School of Medicine in St. Louis, MO. Written informed consent to participate in this study was provided by the participants' legal guardian/next of kin. Written informed consent was obtained from the minor(s)' legal guardian/next of kin for the publication of any potentially identifiable images or data included in this article.

## Author Contributions

EB and AS recruited patients, collected specimens, ran samples, and collected data. SS performed the statistically analysis. EB wrote the manuscript. All authors helped to design the study and edit the manuscript.

### Conflict of Interest

The authors declare that the research was conducted in the absence of any commercial or financial relationships that could be construed as a potential conflict of interest.
